# Vitamin D supplementation in a post-pandemic era: A narrative review

**DOI:** 10.4102/safp.v65i1.5752

**Published:** 2023-10-17

**Authors:** Pheagane G. Bopape, Chrisna Wagenaar, Madan Poka, Elmien Bronkhorst

**Affiliations:** 1 Department of Clinical Pharmacy, Faculty of Health Sciences, School of Pharmacy, Sefako Makgatho Health Sciences University, Pretoria, South Africa; 2 Department of Pharmaceutical Sciences, School of Pharmacy, Sefako Makgatho Health Sciences University, Pretoria, South Africa

**Keywords:** vitamin D, COVID-19, chronic diseases, deficiency, calcifediol, cholecalciferol

## Abstract

Vitamin D is a fat-soluble molecule referring to the different isoforms, ergocalciferol (D2) and cholecalciferol (D3). Its physiological functions include increasing calcium serum concentrations. 25-hydroxyvitamin D_3_ (25(OH)D) (Calcifediol), a non-active, circulating instant precursor is seen as a pre-hormone. Studies have shown that a deficiency in calcifediol is related to chronic conditions such as cardiovascular, musculoskeletal, immune system, neurological, and anti-neoplastic functions. Vitamin D supplementation has shown its benefit as prophylaxis and treatment during the coronavirus disease 2019 (COVID-19) pandemic and an increase in the prescribing of vitamin D supplementation has been observed. The intention of this review article is to provide guidance on the recommended dosage regimen as a prophylactic measure during COVID-19 and its use as a supplement in general. From this review article, it is clear that vitamin D has an important role to play not only in COVID-19 but also in various other health aspects of the human body.

**Contribution:**

This review article highlighted the role of vitamin D in managing vitamin D deficiency and its role as a supplement in the management of respiratory tract infections, especially COVID-19. This overview can assist physicians in optimising healthcare by optimised dosing recommendations and indications.

## Introduction

Vitamin D refers to the different isoforms, ergocalciferol (D_2_) and cholecalciferol (D_3_), resulting from the non-enzymatic reaction utilising ultraviolet B (UVB) light in a thermo-sensitive process.^1^ Vitamin D_2_ and D_3_ are derived from food, sun exposure and supplements. They remain inactive until activated by enzymatic hydroxylation in the liver and kidneys.^2^

Vitamin D is a fat-soluble molecule, classified as a member of the steroid hormone family, which is dissolved in dietary fat and needs to be emulsified by bile salts before absorption. The bioavailabilty of supplements depends on the food supplement vehicle or lipid composition of the formulation. An increase in the bioavailability of the vitamin D formulation is demonstrated in natural oils like peanut, soybean, corn, sesame and olive oil.^3^

Vitamin D receptors (VDR) positioned in the liver, intestines, bones, parathyroid gland and kidneys are the binding sites for the active form of vitamin D. This maintains the body’s calcium-phosphate homeostasis.^4^ A decrease in blood calcium levels causes the parathyroid gland to synthesise parathyroid hormone (PTH), which increases the resorption of calcium in the kidney tubules. It causes the maturation of pre-osteoclasts, resulting in the release of collagenases and hydrochloric acid, which dissolves bones leading to the release of phosphorus and calcium in the circulation.^5^ During vitamin D deficiency, only a maximum of 15% of calcium is absorbed from diet causing a decreased serum calcium concentration, and disrupting bone homeostasis.^5^

**FIGURE 1:**
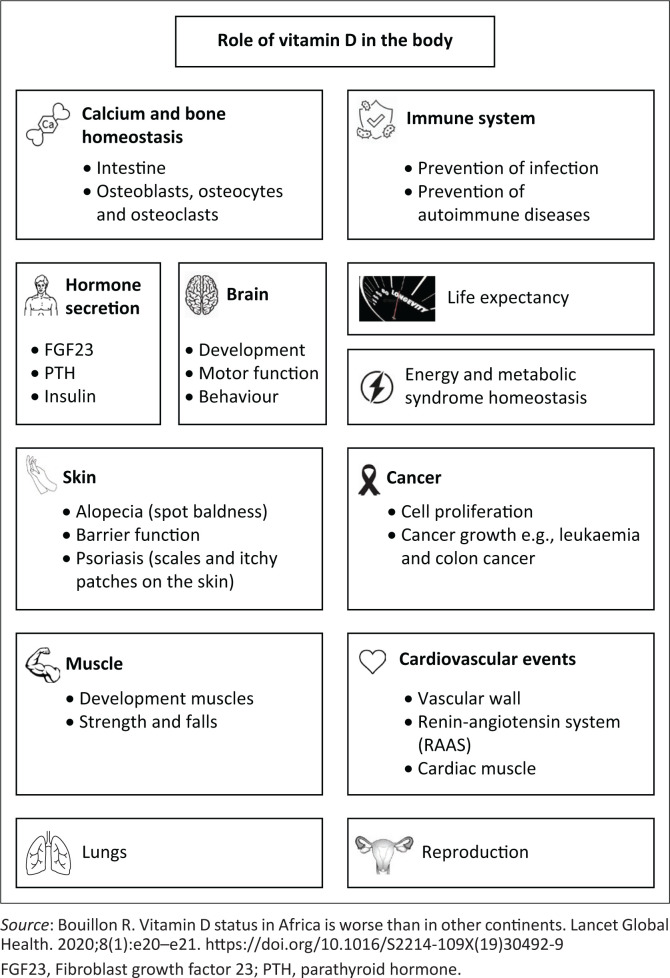
Summarised functions of vitamin D in the endocrine system and targeted tissues.

Vitamin D is an essential co-factor for the absorption of calcium in the intestines. Vitamin D plays a role in many physiological aspects such as increasing calcium serum concentration through renal and intestinal calcium absorption and paracellular calcium transport. It is necessary for healthy cardiovascular, musculoskeletal, immune system and neurological functions.^6^ Figure 1^7^ summarises the functions of vitamin D in the endocrine system and the targeted tissues. Vitamin D has crucial genetic and epigenetics functions,^8^ regulating target genes and providing beneficial effects such as increased expression of genes for a variety of biological pathways. These functions are linked to cardiovascular diseases, autoimmune disorders and cancer.^8,9^

Vitamin D_2_ and D_3_ are metabolised similarly; however, vitamin D_2_ supplementation shows lower efficacy than vitamin D_3_.^7^ A study that measured serum calcifediol in 33 participants by Heaney et al. indicates that vitamin D_3_ was 56% – 87% more potent at raising serum calcifediol levels with area under the curve levels 84 d(AUC_84_) of 1366 ng.d/mL for vitamin D_2_ and 2136 ng.d/mL for vitamin D_3_ when both supplements were given at a dose of 50 000 IU weekly.^10^ Without exposure to sunlight or UVB radiation, diet alone will not provide the requirements for calcitriol, which is the hormonally active metabolite of cholecalciferol, requiring vitamin D supplementation.^7^

## Cholecalciferol, ergocalciferol and calcifediol and rationale for utilisation

Ergocalciferol is primarily less stable and a synthetic product that is not greater in potency per microgram dose compared to cholecalciferol. The cholecalciferol metabolite, ergocalciferol is found in substantial quantities in circulation, while the calcitriol hormone upregulates the active transport of calcium from the gut, and suppresses the secretion of the PTH.^1^ Both cholecalciferol and ergocalciferol are used as supplements during vitamin D deficiency and the choice between the two depends on practical reasons and preference. Ergocalciferol is used predominantly in North America, whereas cholecalciferol is the popular choice in Europe. Research shows similar potency with daily use, but with intermittent use, ergocalciferol is less efficient.^9^ Calcifediol results from the hydroxylation of cholecalciferol at its carbon-25 position, forming a 25-hydroxy-vitamin D3 molecule specified as calcifediol or calcidiol.^9^ Calciferol is the preferred choice of treatment, as it has a higher bioavailability, being absorbed through the vena porta compared to cholecalciferol’s more complex uptake through the lymph.^11^ Calcifediol is not converted in the liver, leading to a linear relationship between dosage and serum concentration.^12^ Calcifediol is more potent; it has a very long half-life (2–3 weeks) and has a higher rate of intestinal absorption, thus a lower dose is required.^13^

Cholecalciferol was deemed to be the preferred form of vitamin D in the most widely accepted and internationally recognised therapeutic guidelines because it has more scientific data supporting its effectiveness in treating musculoskeletal problems than calcifediol.^9^ A study analysing the prescribing patterns of vitamin D among clinical practitioners during the coronavirus disease 2019 (COVID-19) pandemic, which included 4440 practising clinicians indicated that a large number of these prescriptions were found to be in Asia and then followed by Europe.^14^ Additionally, it was noted that compared to medical professionals who would recommend vitamin D for prophylaxis, about 72.8% of general practitioners in these areas would prescribe it for the treatment of COVID-19.^14^ The requirement for the supplementation of vitamin D for all South African adults is still being reviewed. Prescribing of vitamin D in public health is difficult to quantify because clinics, community health centres and public hospitals do not keep records electronically, which makes it difficult to link the consumption nationally.^15^ The prescribing patterns of vitamin D in a private hospital in South Africa (SA) indicated that women between the ages of 50–59 years had the most written prescriptions. These results could be explained by the fact that women have lower bone densities than males and are more likely to sustain fractures from falls as a result of osteoporosis, particularly in the years after menopause.^15^ Vitamin D deficiency-related osteoporosis and osteopenia are not uncommon; this deficiency seems to be more prevalent in women but can be managed with supplementation.^15^

## Prevalence of vitamin D deficiency

Vitamin D deficiency impacts about 5% of the United States population, as indicated by data from the National Health and Nutrition Examination Survey 2011–2014. In Europe, the prevalence is higher, with approximately 14% of individuals being affected, as evidenced by findings from 11 randomised control trials. Moreover, a significant proportion of the population in Middle Eastern and Gulf states experience this deficiency.^16^

In Africa, a high prevalence of an average of one in five people living in Africa had a low calcifediol concentration of less than 30 nmol/L mark; three in ten with a calcifediol concentration of less than 50 nmol/L, and three in every five with a calcifediol concentration of less than 75 nmol/L^17^. Although not a lot of information on the status of vitamin D in children is available. A study done in 2022 in SA showed that 7.6% of school children in a socioeconomically deprived area in Cape Town had a vitamin D deficiency.^18^ A systematic review and meta-analysis that identified 1693 studies, whereby 130 of these studies had 21 676 participants from 23 African countries, found that the prevalence of vitamin D deficiency in African populations is significantly high, with on average, one in five people living in Africa having low calcifediol concentration of less than 30 nmol/L. The study concluded that the vitamin D deficiency prevalence varied by region in Africa, with the highest being reported in Northern African countries and SA.^17^ A study done in 744 infants from the Drakenstein Child Health Study, aged 6 years to 10 years old, showed a prevalence of 81% vitamin D deficiency (< 50 nmol/L).^18^ In the South African adult population, various studies have shown a high prevalence of vitamin D deficiency. For instance, a study by Martineau et al. conducted in Cape Town, South Africa, which investigated the reciprocal seasonal variation in vitamin D status and tuberculosis, found that there was a high prevalence of vitamin D deficiency in its study population whereby 62.7% presented with serum calcifediol levels below 50 nmol/L.^19,20^ Chutterpaul et al. found that vitamin D deficiency and insufficiency was present in 27% and 38%, respectively, in a study conducted on the prevalence of vitamin D deficiency in older South Africans with and without hip fractures and the effects of age, body weight, ethnicity and functional status.^21^

## Consequences of vitamin D deficiency

The resulting hypovitaminosis D increases the occurrence and severity of multiple age-related diseases, such as oxidative stress-associated metabolic disorders, like osteoporosis, insulin resistance, memory disorders among others^7^ and osteomalacia.^22,23^ Children with vitamin D deficiency are not only at risk of developing rickets and growth impairments, but vitamin D deficiency has been linked to multiple adverse child health diseases such as allergies, respiratory tract infections and asthma.^18^ Increasing evidence indicates that the consequences of vitamin D deficiency in the early developmental stages of life may also proceed into adulthood, where there is a correlation between vitamin D deficiency and infections, including COVID-19, cardiovascular disease or cancer.^24^ On the contrary, Manson et al. found that vitamin D supplementation did not result in a lower incidence of invasive cancer or cardiovascular diseases.^25^ Furthermore, children using antiepileptic medication require supplementation with vitamin D, because of the possible destruction of vitamin D as a side-effect of antiepileptic treatment.^26^

## Causes and risk factors for developing vitamin D deficiency

Various factors affecting the synthesis of vitamin D can cause deficiency, including those that can alter sufficient exposure to the sun (necessary to produce vitamin D3). Gender plays a role, and the consumption of food rich in vitamin D (fatty ocean fish, eggs, yellow cheese or milk).^9^ Apart from a multitude of benefits provided by breastfeeding, breastmilk has been shown to contain an inadequate amount of vitamin D levels which can put breastfeeding infants at risk.^27^ Aspects affecting the endogenous synthesis of vitamin D include obesity and low levels of physical activity, pregnancy, small children or infants, as well as age, skin colour and physiological condition.^28^ Dark-skinned individuals living in moderate climates and people experiencing inadequate sunlight exposure due to religious, cultural or other personal reasons are at risk.^28^ Epidemiological and laboratory evidence in recent years demonstrated that vitamin D deficiency is linked to the onset and progression of various chronic diseases.^7,18,29^

## Treatment options and dosages

The initial major intervention is aimed at eradicating severe vitamin D deficiency in individuals, which can be classified as less than 30 nmol/L. Oral supplementation with calciferol is the treatment of choice for deficiencies.^28^ Dietary intake accounts for a significantly low total vitamin D supply; nonetheless it has been substantiated to be a trustworthy source of vitamin D.^30^

The European Food Safety Authority (EFSA) has described a sufficient intake for vitamin D to be 400 IU/day for infants aged 7–11 months. The intake for adults, children aged 1–17 years, pregnant, and lactating women has been recommended to be 600 IU/day.^31^ The individual participant data (IPD) documented that a vitamin D intake of about 30 µg (1200 IU) per day is required to achieve a serum calcifediol concentration of e" 50 nmol/L in 97.5% of the population.^32^ Doses of 50 000 IU vitamin D_3_ administered weekly have been advocated for the correction of vitamin D deficiency; however, insufficient data to make firm recommendations are available.^27^ Du Plessis states that there are some groups of people that need a higher dose of vitamin D of 6000 IU – 10 000 IU daily as an initial dose proceeded by a maintenance dose of 3000 IU – 6000 IU daily to obtain adequate calcifediol levels in the blood.^33^ These groups include obese patients, patients with malabsorption syndromes and those taking medication accelerating vitamin D metabolism due to increased hepatic catabolism because of the drugs inducing P450-enzyme activity. These drugs include carbamazepine, phenytoin, phenobarbital, rifampicin, isoniazid, theophylline and oxcarbazepine. The article further recommended that these patients have a follow-up calcifediol test done after 3 months to 4 months after initiating the treatment. If the patients are compliant with the treatment, and there is no increase found in the calcifediol levels, coeliac disease or occult cystic fibrosis might be considered. Some patients might have normal or upper limits of the normal range of calcium and increased PTH levels. This might be because of patients having a vitamin D deficiency as well as primary hyperparathyroidism. In these patients, vitamin D must be administered as a supplement to prevent bone loss, but caution must be taken because there is a slight chance that hypercalcaemia and hypercalciuria might develop. Among individuals with granulomatous disease that needs vitamin D supplement, caution must be taken that the vitamin D levels should not rise above 30 nmol/L as it might also cause hypercalciuria and hypercalcaemia.^33^ Patients suffering from osteoporosis also need to take in more vitamin D and calcium. They can do this by supplementing vitamin D with or without calcium supplements. The usual dose is 200 IU to 500 IU per 500 mg to 600 mg of calcium.^34^ In patients with hypoparathyroidism, calcitriol should be administered as an initial dose of 10 IU to 20 IU daily and a maintenance dose of 20 IU – 80 IU daily. This low dose is due to the fact that people with hypoparathyroidism have a decreased PTH level, and this deficiency might lead to the inability to regulate the absorption of calcium and phosphate, which may lead to hypercalcaemia or an imbalance of calcium-phosphate balance.^35^ In patients suffering from rickets, the chosen treatment usually consists of daily doses of vitamin D_2_ and D_3_. The dosages are different according to different age groups. Infants of an age younger than 1 month old can be given 1000 IU (daily for as long as 3 months), and then a maintenance dose of 400 IU daily can be administered. Those aged 1 month to 12 months old can receive 1000 IU to 1200 IU daily for as long as 3 months and then a dose of 400 IU (10 mcg) daily as maintenance dose. Children of 1 year to 12 years can be administered 2000 IU to 6000 IU daily for 3 months and then a maintenance dose of 600 IU daily. Children older than 12 years of age can get an initial dose of 6000 IU daily for 3 months and a maintenance dose of 600 IU daily.^27^

## Vitamin D action against respiratory tract infections in relation to COVID-19

During the COVID-19 pandemic, vitamin D among other nutritional supplements such as calcium, and vitamin C was commonly prescribed for the management of COVID-19.^36^ Vitamin D has shown benefits in reducing the severity of COVID-19 and its benefits as prophylaxis for COVID-19 in many studies.^20,36,37,38^

People receiving vitamin D supplementation present with less risk of acquiring respiratory tract infections, as reported by a meta-analysis and systemic review.^20^ A study conducted by the Council for Responsible Nutrition that took place during the 2020 COVID-19 pandemic reported an increase in the use of supplements during this period. About 37% of the 554 participants of the survey reported that they used vitamin D as one of the top 10 supplements. An increase in COVID-19 mortality associated with vitamin D deficiency was cited as the reason for the increased use of vitamin D in a similar survey about nutritional supplements to improve immunity. Less severe COVID-19 infection was found in elderly patients who received vitamin D supplementation and significantly low levels of serum vitamin D were associated with poor disease prognosis.^39,40^ Another survey reported less hospital admission and less sleep disturbances in patients who received prophylactic vitamin C and D for longer than 2 weeks. Furthermore, high initial vitamin D doses to achieve adequate serum levels resulted in less severity of the disease.^41^

**FIGURE 2:**
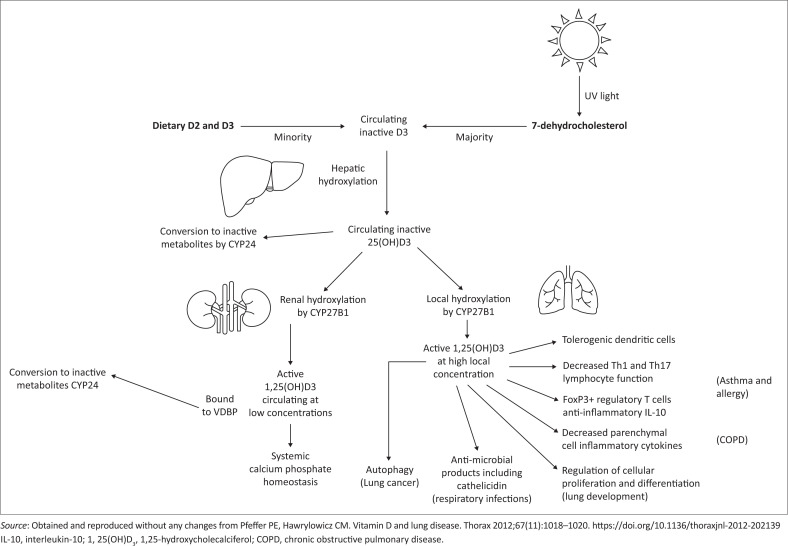
The different mechanisms in which vitamin D aids in resolving respiratory tract infections.

Vitamin D deficiency causes the lungs to lose epithelial integrity, which makes it more prone to inflammatory pathologies and processes.^42^ The use of vitamin D has been recommended prior to and during the infection of COVID-19 as there are associations made with vitamin D and lower severity of the disease.^41^ Figure 2^44^ illustrates the different mechanisms in which vitamin D aids in resolving respiratory tract infections. A possible mechanism is through the epithelial cells in human airways, which express an essential VDR and secrete vitamin D, resulting in increased expression of antimicrobial peptides (such as defensin beta-4 and cathelicidin).^42^ Th1 responses are enhanced in severe COVID-19, which is thought to be a factor in pathogenic hyper-inflammation.^43^ In animal models of pneumonia and pneumonitis, vitamin D has been shown to continue to reduce the inflammatory cytokine response to infections in T cells and macrophages.^42^

## Vitamin D toxicity

Vitamin D toxicity occurs during exposure to high doses of vitamin D, which results of elevated calcifediol (> 375 nmol/L), and usually normal or slightly increased calcitriol concentration.^45^ The primary role of vitamin D is to improve the intestinal absorption of calcium, However, hypercalcaemia and hypercalciuria can occur if vitamin D is used inappropriately.^46^ The presentation of toxicity can be symptomatic, including neuropsychiatric effects (confusion, psychosis, stupor and coma), gastrointestinal (abdominal pain, nausea and vomiting) and cardiovascular symptoms (ST-segment elevation, QT-segment interval shortening and hypertension) or it can be asymptomatic.^47^ Management of toxicity requires discontinued use of the supplement and reduced ingestion of dietary calcium. Administration of isotonic sodium chloride solution to manage dehydration and administration of glucocorticoids to reduce calcium absorption from the intestines may be indicated.^45^

## Conclusion

Vitamin D has an important role to play not only in COVID-19 but also in various health aspects of the human body. Supplementing with vitamin D can curb deficiencies causing osteomalacia, osteoporosis, hypoparathyroidism, vitamin D-resistant rickets and familial hypophosphataemia among a myriad of other conditions. During the COVID-19 pandemic, it also emerged as a prophylactic and treatment option. Coronavirus disease 2019 is a respiratory infectious disease caused by the severe acute respiratory syndrome coronavirus 2 (SARS-CoV-2). Vitamin D deficiency increases the risk of acute respiratory distress syndrome and lung injury; additionally, it contributes to the risk of developing cardiovascular events, diabetes and associated comorbidities, which are the primary factors that lead to serious clinical problems in COVID-19.
